# Palliative Performance Scale: cross cultural adaptation and psychometric validation for Polish hospice setting

**DOI:** 10.1186/s12904-020-00563-8

**Published:** 2020-04-22

**Authors:** Tomasz Dzierżanowski, Tomasz Gradalski, Michael Kozlowski

**Affiliations:** 1 Laboratory of Palliative Medicine, Department of Social Medicine and Public Health, Medical University of Warsaw, Warsaw, Poland; 2 St Lazarus Hospice, 31-831 Krakow, Fatimska, 17 Krakow, Poland; 3 Clinic of Pain Treatment and Palliative Care, Chair of Internal Medicine and Geriatrics, Jagiellonian University Medical College, Krakow, Poland

**Keywords:** Performance status, Scale, Validation, Hospice, Palliative

## Abstract

**Background:**

Measuring functional status in palliative care may help clinicians to assess a patient’s prognosis, recommend adequate therapy, avoid futile or aggressive medical care, consider hospice referral, and evaluate provided rehabilitation outcomes. An optimized, widely used, and validated tool is preferable. The Palliative Performance Scale Version 2 (PPSv2) is currently one of the most commonly used performance scales in palliative settings. The aim of this study is the psychometric validation process of a Polish translation of this tool (PPSv2-Polish).

**Methods:**

Two hundred patients admitted to a free-standing hospice were evaluated twice, on the first and third day, for test-retest reliability. In the first evaluation, two different care providers independently evaluated the same patient to establish inter-rater reliability values. PPSv2-Polish was evaluated simultaneously with the Karnofsky Performance Score (KPS), Eastern Cooperative Oncology Group (ECOG) Performance Status (ECOG PS), and Barthel Activities of Daily Living (ADL) Index, to determine its construct validity.

**Results:**

A high level of full agreement between test and retest was seen (63%), and a good intra-class correlation coefficient of 0.85 (*P* < 0.0001) was achieved. Excellent agreement between raters was observed when using PPSv2-Polish (Cohen’s kappa 0.91; *P* < 0.0001). Satisfactory correlations with the KPS and good correlations with ECOG PS and Barthel ADL were noticed. Persons who had shorter prognoses and were predominantly bedridden also had lower scores measured by the PPSv2-Polish, KPS and Barthel ADL. A strong correlation of 0.77 between PPSv2-Polish scores and survival time was noted (*P* < 0.0001). Moderate survival correlations were seen between KPS, ECOG PS, and Barthel ADL of 0.41; − 0.62; and 0.58, respectively (*P* < 0.0001).

**Conclusion:**

PPSv2-Polish is a valid and reliable tool measuring performance status in a hospice population and can be used in daily clinical practice in palliative care and research.

## Background

Functional decline, in addition to symptom burden, results in increased dependency on others and negatively affects a patient’s quality of life. Most people want to remain symptom-free and to maintain as much independence as possible until the latest phases of life. The importance of measuring functional status in palliative care is incontestable. It may help clinicians to assess a patient’s prognosis, recommend further oncological therapy, avoid futile, aggressive medical care, consider hospice referral and evaluate provided rehabilitation outcomes. All measuring tools may have disadvantages, and an optimal one, which may also be used in monitoring the outcomes of physical therapy, specifically in palliative care, is lacking [1]. The Palliative Performance Scale version 2 (PPSv2) is currently one of the most commonly used performance scales in palliative settings. It was developed by the Victoria Hospice Society as a modification of the Karnofsky Performance Scale in 1996 [2] and validated later [3]. It has also been demonstrated that PPSv2 correlates with patients’ survival time [4]. Although occasionally used in Poland, it has not been validated yet. It is reasonable to disseminate knowledge among professionals and promote its use through cultural adaptation.

## Methods

### Aim

The aim of this study is the cross cultural adaptation and psychometric validation of a Polish translation of PPSv2 tool (PPSv2-Polish).

### Design

We obtained agreement and guidelines for the translation process from the Victoria Hospice Society, the owner of the tool.

The tool translation and back-translation were done in cooperation of three palliative care centers: St. Lazarus Hospice in Krakow, Medical University of Warsaw (Poland), and Jagiellonian University Medical College, Krakow, Poland.

The testing and retesting of PPSv2-Polish was performed by the hospice care team at St. Lazarus Hospice in Krakow (Poland).

Patient characteristics, including gender, age, primary diagnosis, and disease stage based on the Gold Standard Framework needs based coding (GSF; see Table 1) [5] assessed by the attending physician, were obtained from medical records. All participants were evaluated twice, on the first and third day, for test-retest reliability by the same member of the hospice care team at St. Lazarus Hospice in Krakow (Poland) (a trained and experienced palliative care nurse, psychologist, or physiotherapist), who cared for the patient. Additionally, on the first evaluation, two different care providers (each time by different types of professionals) independently evaluated the same patient to establish inter-rater reliability values. Each patient’s evaluation encompassed the PPSv2-Polish, which was compared simultaneously with 3 additional performance scales, considered the gold standard [6], to accomplish its external construct validity.

**Table 1:** GSF Needs Based Coding

GSF Code	Disease progression	Expected prognosis
A	Stable from diagnosis	Years
B	Unstable / advanced disease	Months
C	Deteriorating	Weeks
D	Final days	Days

*Abbreviations*: *GSF* Gold Standard Framework

### Participants

Among 223 patients consecutively admitted to an in-patient hospice (between September 1st and November 30th 2019), 200 (aged ≥18 years), who were Polish native speakers whose performance status was unlikely to change significantly during the nearest 3 days according to the attending physician, were recruited and enrolled in the study.

### Measures

#### Palliative performance scale version 2 (PPSv2)

The PPS provides a functional assessment of a patient’s ambulation, activity level, evidence of disease, self-care, food/fluids intake, and level of consciousness. The PPS has 11 categories, from 100% (full mobile and healthy) to 0% (dead) in decrements of 10%. In 2006, PPS version 2 (PPSv2) was introduced after clarification of instructions for its use [7].

#### PPSv2-polish translation process

**Fig. 1:**
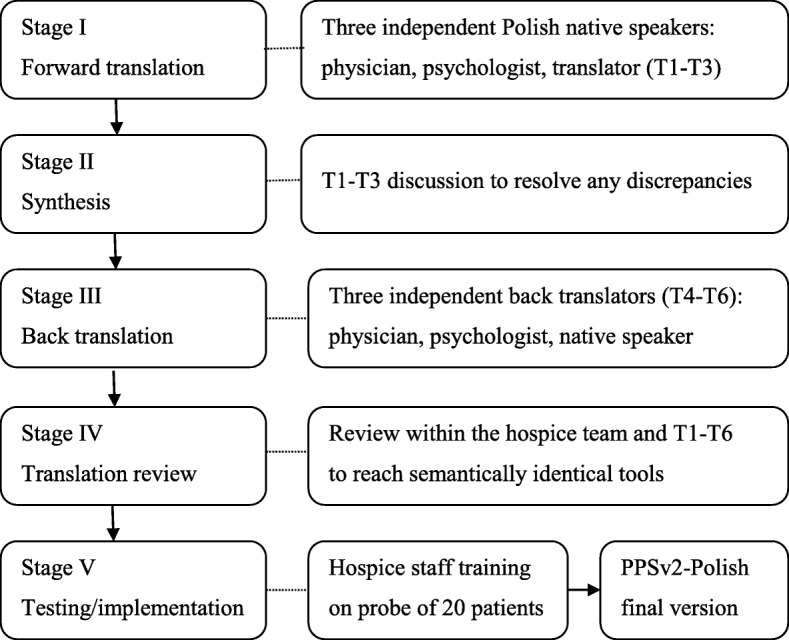
Graphical representation of translation process of PPSv2-Polish

A modified combined translation technique [8] of PPSv2 into Polish was applied, which consisted of 1) independent forward translations by a physician, a psychologist and a Polish native speaker, 2) team discussion on identified differences between these 3 versions until agreement, 3) independent backward translations by a physician, a psychologist and a native English speaker, and 4) second team discussion on any differences between the original and back-translated versions until all agreed that the two versions were semantically identical (Fig. 1).

This method followed the Victoria Hospice translation guidelines sent to the authors. The discussed back-translated version was then preliminarily tested in the hospice on 20 patients to obtain the final version of the PPSv2 (PPSv2-Polish – see Additional file 1). The implementation process encompassed education of the medical staff participating in this study during one training session, based on the Victoria Hospice Society instructions sent to the authors, giving the opportunity to get feedback from the team members.

#### Karnofsky performance score (KPS)

The KPS ranking is an 11-point scale and runs from 100% - perfect health, to 0% - dead. While first published in 1948 [9] to evaluate the ability to survive chemotherapy for cancer, it has recently undergone several evaluation adjustments [10]. The KPS provides great consistency of ratings by different oncology professionals [11]. It may also serve as a life survival predictor [12]. Hovewer, it has not been validated in Poland yet.

#### Eastern cooperative oncology group (ECOG) performance status (ECOG PS)

This scale, also called the World Health Organization (WHO) score, was published in its current form by ECOG in 1982 [13] to assess a patient’s level of functioning in terms of the ability to care for himself, daily activity, and physical ability, in order to measure the impact of the disease/treatment on performance status. It has a good intra and interobserver agreement in cancer patients’ performance status assessment [14]. It consists of 6 categories, from 0 - fully active, to 5 - dead, and is simpler to use, may be clinically preferable in comparison to the KPS, [15] and is widely used in the literature, yet not validated in Poland.

#### Barthel activities of daily living (ADL) index

This “simple index of independence” was published in 1965 for measuring the improvement during rehabilitation of the chronically ill" [16]. The original version was modified in 1988 to a 20 point scale that measures in increments of 1 point: from 0 - fully dependent, to 20 - fully independent [17]. The final score can be multiplied by 5 to obtain a 100 point score, and it is proposed that scores of 0–20 indicate “total” dependency, 21-60 indicate “severe” dependency, 61–90 indicate “moderate” dependency, and 91–99 indicates “slight” dependency. It is already widely used as the measurement of daily living activities and has become a standard measure of physical disability in practice [18]. Ten categories are assessed: feeding, grooming, bathing, dressing, toilet use, presence of fecal incontinence, presence of urinary incontinence, transfers (e.g., moving from wheelchair to bed), walking on an even surface (or propelling a wheelchair if unable to walk), and ascending and descending stairs. This scale has been widely used, however, has not yet been adapted in Poland.

### Statistical analysis

We summarized the baseline demographics using descriptive statistics and medians with interquartile ranges (IQR) in non-normally (according to Shapiro-Wilk test) distributed ordinal quantitative data. A Wilcoxon signed-rank test was used to compare test-retest of ordinal data in one sample in test-retest and inter-rater comparison. Non-parametric data within subgroups of patients were compared using a Mann-Whitney U test. The strength of the relationship between the test-retest variables, and between the tools scores and survival time were calculated with the Spearman’s rank correlation coefficient. The inter-rater reliability was estimated using Cohen’s kappa statistics. Data were analyzed using STATISTICA 13.0 (TIBCO Software Inc. 2017) data analysis software. A *P*-value of < 0.05 was considered as the level for statistical significance. The sample size for this survey was based on general guidelines for conducting qualitative research [19] As there are no absolute rules for the sample size needed to validate a questionnaire a fair size of 200 patients was chosen.

## Results

In all cases the hospice staff were able to complete the evaluation according to the study protocol (no missing values, a response rate of 100%), and they assessed the tool as easy to use and not excessively time consuming. This indicates that the PPSv2-Polish appeared a feasible and acceptable assessment tool in their practice. At the beginning of implementation, in the training phase of this study, two out of six team members made observations that the 5-steps of PPSv2-Polish assessment was a bit prolonged, in comparison with 1-step assessment of KPS or ECOG PS. All participating staff emphasized the need for observation of a patient for a reasonable period of time to accurately asses his or her true “capable” functions based on the “observed” ones, during the day shift.

The majority of patients were aged, had advanced cancer, with short (weeks) prognosis according to GSF, and finally died at the hospice (Table 2).

**Table 2:** Patient characteristics and description

Parameter	*n* = 200	%
Median age in years (IQR)	72.5	(16.0)
Females	144	72.0
Primary cancer tumor site		
Digestive	34	17.0
Respiratory	35	17.5
Genitourinary	22	11.0
Breast	21	10.5
Others	67	33.5
Nonmalignant diseases	21	10.5
Stage – prognosis (GSF)		
B – Stable/months	32	16.0
C – Progressing/weeks	141	70.5
D – Last days	27	13.5
ECOG PS		
1	3	1.5
2	23	11.5
3	41	20.5
4	133	66.5
Died		
In 1–30 days	117	58.5
In > 30 days	58	29.0
Discharged	25	12.5

*Abbreviations*: *IQR* interquartile range, *GSF* Gold Standards Framework, *ECOG PS* Eastern Cooperative Oncology Group Performance Status,

### Test-retest reliability

The median PPSv2-Polish value within the first measurement was 30 (IQR 10), which correlated with the data obtained 2 days later (median 30; IQR 20) by the same care provider. We achieved a high level of full agreement between test and retest (63%) and a good intra-class correlation coefficient of 0.85 (*P* < 0.0001).

### Inter-rater reliability

An excellent PPSv2-Polish agreement between raters was observed (Cohen’s kappa 0.91; *P* < 0.0001). A high level (94–99%) of full agreement between raters was observed, with the exception of ECOG PS, where this agreement was not achieved (Table 3).

**Table 3:** Inter-rater agreement of scales used in the study

Scales	Rater #1		Rater #2		% full agreement	*P*
	Median	IQR	Median	IQR		
PPSv2-Polish	30.0	10.0	30.0	10.0	94.0	0.13
KPS	45.0	10.0	40.0	10.0	99.0	0.18
ECOG PS	4.0	1.0	4.0	10.0	95.5	0.54
Barthel ADL	5.0	22.5	5.0	22.5	83.0	0.001

*P* Wilcoxon signed-rank test,

*Abbreviations*: *IQR* interquartile range, *PPSv2-Polish* Polish translation of the Palliative Performance Scale Version 2, *KPS* Karnofsky Performance Score, *ECOG PS* Eastern Cooperative Oncology Group Performance Status, *ADL* Activities of Daily Living Index.

### Criterion validity

Satisfactory correlations with the KPS and good correlation with ECOG PS and Barthel ADL were noticed (Table 4).

**Table 4:** Correlations between the scales used in the study

	KPS	ECOG	Barthel ADL
PPSv2-Polish	0.69*	−0.81*	0.75*
KPS		−0.57*	0.68*
ECOG PS			−0.73*

* Spearman’s Rho *P* < 0.001, *Abbreviations*: *PPSv2-Polish* Polish translation of the Palliative Performance Scale Version 2, *KPS* Karnofsky Performance Score, *ECOG PS* Eastern Cooperative Oncology Group Performance Status, *ADL* Activities of Daily Living

### Known-group validity

Persons who had shorter prognosis and were predominantly bedridden also had lower scores measured by the PPSv2-Polish, KPS and Barthel ADL (Table 5).

**Table 5:** Responsiveness of the scales

Scales	GSF A-B *n* = 32		GSF C-D *n* = 168		*P*	ECOG 1–2 *n* = 26		ECOG 3–4 *n* = 174		*P*
	Median	IQR	Median	IQR		Median	IQR	Median	IQR	
PPSv2-Polish	50.0	15.0	30.0	10.0	*	60.0	20.0	30.0	10.0	*
KPS	60.0	15.0	40.0	10.0	*	60.0	10.0	40.0	10.0	*
Barthel ADL	42.5	40.0	5.0	7.5	*	45.0	60.0	5.0	10.0	*

Median scores of each scale obtained within the known subgroups of longer (GSF A-B) and shorter (GSF C-D) prognosis and also within higher (ECOG PS 1–2) and lower (ECOG PS 3–4) performance status. * *P* < 0.00001, Mann-Whitney U test

*Abbreviations*: *GSF* Gold Standard Framework, *ECOG* Eastern Cooperative Oncology Group, *PPSv2-Polish* Polish translation of the Palliative Performance Scale Version 2, *KPS* Karnofsky Performance Score, *ECOG PS* Eastern Cooperative Oncology Group Performance Status, *ADL* Activities of Daily Living Index.

### Survival estimation

A strong correlation between each category assessed in PPSv2-Polish and survival time was noted (0.77; *P* < 0.0001). Median survival time (IQR) in GSF B, C and D group was respectively 37 (21.3), 23 (13.0) and 12 (23.5) days. Moderate survival correlations were observed between KPS, ECOG PS, and Barthel ADL Index scores (0.41, − 0.62, and 0.58, respectively; *P* < 0.0001).

## Discussion

The PPSv2-Polish which was created with a combined translation technique appears to be a valuable clinical assessment tool in the hospice setting within the Polish population of cancer patients. The translation and implementation process and training of experienced palliative care medical staff proceeded without any particular difficulties. The team members reported that the PPSv2-Polish tool was clear and easy, although a bit time consuming to use in daily practice, and it required observation of a patient for a significant period of time (e.g. through a whole shift) to assess the potential capability of evaluated functions. When comparing various performance tools, it appears that no one is statistically superior to others in terms of inter-rater reliability [20]. The ECOG PS or KPS are often used in determining eligibility for clinical trials. However, there could be a substantial disagreement in the assessment of performance status between oncologists, even when using as simple a tool as the ECOG PS [21]. Patients’ age, preferences and socio-economic background may also influence the assessment. Numerous studies confirmed good correlations of KPS, ECOG PS, and PPS tools, and meta-analysis favors KPS as descriptively better [20]. Authors of the original version of the PPSv2 noticed that in contrary to the KPS, this scale does not focus on the need for hospitalization (which is of poor definability and does not help in defining performance) but instead assesses food/fluid intake and level of consciousness [2]. The ambiguity of the KPS assessment when patients were bedridden (KPS ≤ 40%) led to scale modification in Australia [22]. PPSv2 usage assumes a 5-step assessment, which can be problematic at the beginning, but after training may be more comprehensive and accurate. Both KPS and PPSv2 scales need standardized, appropriate instructions regarding performance evaluation. Compared with the 5-point ECOG PS, the 11-point PPSv2 seems to be a more precise tool, especially for lower performance statuses. This phenomenon was also affirmed in our observations, where the ECOG PS did not achieve significant reliability between the scoring of different types of professionals.

The high level of agreement with a very good correlation in serial scoring by one rater in a 2 days interval appeared better than in another study with 2 weeks between consecutive assessments [3]. The chosen interval of 2 days between assessments allowed for an optimal period not to recall the first scoring, yet not too long to allow changes in performance in most cases.

The excellent inter-rater concordance observed in this study was higher than in an updated meta-analysis recently published [20]. The explanation of this phenomenon partially could be explained by the careful inclusion of rating staff, who attended to patients for several hours daily and had a great understanding of their mobility and functionality [11].

The strength of this study was the high inter-rater agreement each time between different professionals (nurse, psychologist, or physiotherapist) took part in the assessment. There are inconsistencies in the evaluation of a patient’s performance status using the same tool by different types of professionals (doctors rated patients as healthier than nurses using the PPSv2 scale), and it may be explained by different amounts of time spent with the evaluated person [11]. However, even research asistants rated patients simillar to physicians (oncologists or radiation therapists) in one study [23]. Optimally, it should be advised to score patients within interdisciplinary team meetings to gain a more accurate assessment [24].

Our study legitimizes the usage of the PPSv2-Polish in prognostication of patients with advanced cancer, which is in line with previous studies [4, 7]. The strongest correlation between the PPSv2-Polish and survival time among the analysed tools was remarkable, and as to the best of our knowledge it was not published earlier. In another recent study of advanced cancer patients with prognosis in terms of weeks, the PPSv2 assessment was as accurate as subjective clinical survival predictions [25]. The similarity between the subjective prognosis assessment of the attending physician, expressed by GSF staging, and the PPSv2 scoring in our observations legitimizes the good responsiveness of this newly validated tool to patients’ changing prognosis and physical condition. This finding could be explained by the strong relationship between the hospice staff and the patient, as this factor was described to have an impact on accuracy [26].

This study was not without limitations. First, the majority of patients recruited presented a low performance status, were mostly sitting or bedridden, and were not representative for the whole palliative population. Secondly, only in-patient subjects were recruited and most of the unstable patients were excluded due to the test-retest requirements.

## Conclusion

The Polish version of PPSv2 is a valid and reliable tool measuring performance status in a hospice population, which can be used in daily clinical practice in palliative care, research, and prognostication.

## Supplementary information

**Additional file 1.** Polish translation of the Palliative Performance Scale Version 2

## Data Availability

The dataset used and analysed during the current study are available from the corresponding author on reasonable request.

## Abbreviations

PPS: Palliative Performance Scale
PPSv2: Palliative Performance Scale Version 2
PPSv2-Polish: Polish translation of the Palliative Performance Scale Version 2
KPS: Karnofsky Performance Score
ECOG: Eastern Cooperative Oncology Group
ECOG PS: Eastern Cooperative Oncology Group Performance Status
Barthel ADL: Barthel Activities of Daily Living Index
IQR: Interquartile range
GSF: Gold Standard Framework
