# Supplementary material for: Palliative Performance Scale: cross cultural adaptation and psychometric validation for Polish hospice setting
Source: BMC Palliat Care. 2020 Apr 22;19:52. doi: 10.1186/s12904-020-00563-8 (PMC7178730; doi:10.1186/s12904-020-00563-8)
Supplement: Supplementary file 1 — Additional file 1. Polish translation of the Palliative Performance Scale Version 2 [file 12904_2020_563_MOESM1_ESM.pdf]

Palliative Performance Scale version 2 – Polish (PPSv2-Polish)

PPS	mobilność	aktywność i oznaki choroby	samoobsługa	spożywanie
100%	pełna	normalna aktywność i praca<br>brak oznak choroby	pełna	normalne
90%	pełna	normalna aktywność i praca<br>niewielkie oznaki choroby	pełna	normalne
80%	pełna	normalna aktywność z wysiłkiem<br>niewielkie oznaki choroby	pełna	normalne<br>lub ograniczone
70%	ograniczona	normalna praca niemożliwa<br>istotne oznaki choroby	pełna	normalne<br>lub ograniczone
60%	ograniczona	praca domowa / hobby niemożliwe<br>istotne oznaki choroby	okazjonalna pomoc<br>potrzebna	normalne<br>lub ograniczone
50%	przeważnie siedzący/leżący	niezdolny do wykonywania żadnej pracy<br>nasilona choroba	znacząca pomoc<br>wymagana	normalne<br>lub ograniczone
40%	przeważnie w łózku	niezdolny do wykonywania wiekszości aktywności<br>nasilona choroba	głównie pomoc	normalne<br>lub ograniczone
30%	całkowicie leżący	niezdolny do wykonywania żadnej aktywności<br>nasilona choroba	całkowita opieka	normalne<br>lub ograniczone
20%	całkowicie leżący	niezdolny do wykonywania żadnej aktywności<br>nasilona choroba	całkowita opieka	minimalne ilości<br>do łyczków
10%	całkowicie leżący	niezdolny do wykonywania żadnej aktywności<br>nasilona choroba	całkowita opieka	jedynie toaleta<br>jamy ustnej
0%	śmierć	-	-	-

Instrukcja stosowania skali PPS (patrz także definicje pojęć)

1. Wynik PPS uzyskuje się poprzez odczytywanie wzdłuż wersów zawartości poszczególnych poziomów – celem znalezienia "najlepiej odpowiadającego" pacjentowi poziomu, a następnie sprawdzenie przypisanej mu punktacji w % (PPS%).
2. Należy rozpocząć od lewej kolumny, kierować się ku dołowi aż do znalezienia właściwego poziomu mobilności, następnie przejść na tej samej wysokości do następnej kolumny i również kierować się ku dołowi, aż do znalezienia poziomu, w którym przytoczona jest właściwa aktywność/oznaka choroby. Przed określeniem faktycznego wyniku, należy przesuwać się w podobny sposób przez wszystkie kolumny. Tak więc kolumny ulokowane po lewej (znajdujące się bardziej po lewej w stosunku do określonej kolumny) w większym stopniu wpływają na wynik, są "uprzywilejowane".

   Przykład 1. Pacjent spędzający większość dnia siedząc lub leżąc ze względu na zmęczenie spowodowane zaawansowaną chorobą, wymagający znaczącej pomocy aby przejść nawet niewielką odległość lecz mający zachowaną pełną świadomość i normalnie spożywający, powinien być oceniony na poziomie 40%.

   Przykład 2. Pacjent sparaliżowany czterokończynowo, wymagający całkowitej opieki, powinien być oceniony na PPS 30%. Chociaż pacjent ten wysadzony na wózek może być oceniony wstępnie na 50%, ostatecznie jest oceniany na 30%, ponieważ gdyby nie pomoc, od której pacjent jest całkowicie zależny w zakresie przemieszczania, byłу osobą całkowicie leżącą.

   Przykład 3. Jeżeli pacjent z przykładu 2, całkowicie leżący, pomimo paraplegii, byłby zdolny do wykonywania niektórych czynności samoobsługowych, np. przyjmowania posiłków, to wynik PPS byłby wyższy: 40% lub 50%, gdyż pacjent nie wymagałby całkowitej opieki.

3. Wyniki PPS określa się podając wynik zmieniający się jedynie co 10%. Czasem kilka kolumn określa chorego na jednym poziomie z wyjątkiem jednej lub dwóch, które określają stan wyżej lub niżej. Należy wówczas podjąć decyzję w oparciu o „najlepsze dopasowanie". Wybór połowiczny, np. PPS 45%, nie jest prawidłowy. Decyzję, czy wynik 40% czy 50% jest bardziej trafny, należy podjąć w oparciu o ocenę kliniczną oraz „pierwszeństwo" kolumn znajdujących się po lewej stronie.
4. Skala PPS może służyć różnym celom. Po pierwsze, jest narzędziem usprawniającym komunikację dotyczącą aktualnego stanu funkcjonowania pacjenta. Po drugie, może być kryterium wartościującym obciążenie pracą, służyć pomocą w innych ocenach i być wykorzystywana do porównań. Poza tym może ona mieć wartość prognostyczną.

Prawa autorskie © 2001 Victoria Hospice Society

Definiowanie pojęć dotyczących PPS

Jak podano poniżej, niektóre określenia maja podobne znaczenie, a różnice są łatwiej dostrzegalne, gdy czytając każdy wiersz poziomo stara się znaleźć „najlepsze dopasowanie”, wykorzystując wszystkie pięć kolumn.

1. Mobilność
Określenia **„przeważnie siedzący/leżący”**, **„przeważnie w łóżku”** i **„całkowicie leżący”** są w naturalny sposób zbliżone. Subtelne różnice wyjaśnia kolumna samoobsługa. Przykładowo, chory „całkowicie leżący”, określany jest jako PPS 30% albo na skutek znacznego osłabienia, albo na skutek paraliżu, w wyniku którego pacjent nie tylko nie jest w stanie opuścić łózka, ale także nie jest zdolny do samoobsługi. Różnica pomiędzy „przeważnie siedzący/leżący” i „przeważnie w łóżku” jest proporcjonalna do ilości czasu, w którym pacjent jest zdolny do siedzenia vs. musi leżeć.

„Ograniczona mobilność” lokuje się na poziomach PPS 70% i PPS 60%. Wykorzystując sąsiednią kolumnę (aktywność i oznaki choroby), ograniczoną mobilność wiąże się z niemożnością wykonywania normalnej pracy i aktywności zawodowej lub niektórych hobby czy prac domowych. Osoba taka jest w stanie samodzielnie chodzić i przemieszczać się, ale na poziomie PPS 60% potrzebuje okazjonalnej pomocy.

2. Aktywność i oznaki choroby
„Niewielkie”, **„istotne oznaki”** oraz **„ nasilona choroba”** odnoszą się do danych klinicznych lub z wywiadu, wskazujących na stopień progresji. Przykładowo, w raku piersi: wznowa miejscowa będzie oznaczać „niewielkie” oznaki choroby, jedna lub dwie zmiany przerzutowe w płucach lub kościach będą „istotnymi” oznakami, podczas gdy liczne przerzuty w płucach, kościach, wątrobie i mózgu, hiperkalcemia lub inne poważne następstwa nowotworu będą oznaczać „nasiloną” chorobę. Nasilenie może także oznaczać progresję choroby pomimo stosowanej terapii. Stosując skalę PPS w przypadku AIDS, „niewielkie” oznaki wiążą się przejściem HIV w AIDS, „istotne” z progresją osłabienia fizycznego, nowymi lub trudnymi do opanowania objawami lub deficytem komórek w badaniach laboratoryjnych. „Nasilona choroba” odnosi się do jednego lub więcej poważnych powikłań w trakcie lub nawet bez leczenia antyretrowirusowego, antybiotykoterapii, itd.

Powyższe nasilenie choroby jest również oceniane w kontekście możliwości kontynuowania pracy, hobby lub aktywności. Przykładowo, chory może poświęcać mniej czasu na sport rekreacyjny lub skracać dystans spacerów, które jednak mogą być przez chorego kontynuowane, nawet w obliczu zbliżającej się śmierci.

3. Samoobsługa
„Okazjonalna pomoc” oznacza, że pacjent przez większość czasu jest zdolny do samodzielnego opuszczania łóżka, chodzenia, mycia się, korzystania z toalety, chociaż okazjonalnie (np. raz dziennie, kilka razy w tygodniu) wymaga niewielkiej asysty.

„Znacząca pomoc” oznacza konieczność regularnej, codzienne pomocy w powyższych aktywnościach, udzielanych zwykle przez jedna osobę. Przykładowo, chory wymaga pomocy w przejściu do łazienki, ale samodzielnie jest w stanie umyć sobie zęby albo przynajmniej twarz i ręce. Pożywienie choremu często trzeba pokroić na odpowiednie porcje ale tak przygotowany pokarm jest on w stanie samodzielnie przyjmować.

„Głównie pomoc” jest kolejnym etapem ograniczenia samoobsługi. Wykorzystując powyższy przykład, pacjent potrzebuje teraz pomocy przy wstaniu z łóżka, myciu twarzy czy goleniu się, chociaż jest w stanie jeść samodzielnie lub z minimalną pomocą. Ten stan może się zmieniać w ciągu dnia.

„Całkowita opieka” oznacza, że pacjent w ogóle nie jest w stanie samodzielnie jeść, korzystać z toalety. i nie jest zdolny do samoobsługi. W zależności od stanu klinicznego, karmiony może (choć nie musi) żuć i połykać podawany pokarm.

4. Spożywanie
Zmiany dotyczące spożywania pokarmów mogą być łatwe do zaobserwowania w odniesieniu do nawyków żywieniowych danej osoby, oznaczających spożywanie **„normalne”**. Zmniejszenie ilości w stosunku do indywidualnej dla pacjenta normy określane jest jako **„ograniczone”**. **„Minimalne”** odnosi się do przyjmowania bardzo małych objętości pożywienia, zwykle miksowanego lub płynnego, w ilości znacznie mniejszej niż zapotrzebowanie pacjenta.

5. „Poziom świadomości”
„Pełna” oznacza pełną świadomość i orientację z zachowanymi zdolnościami poznawczymi jak myślenie, pamięć, itp. **„Splątanie”** oznacza majaczenie lub otępienie wraz z obniżeniem poziomu świadomości. Może ono być łagodne, średniego lub ciężkiego stopnia, o różnorodnej etiologii. Określenie **„senny”** może dotyczyć osłabienia, efektów podawanych leków, majaczenia lub bliskości śmierci, czasem też stuporu. **„Śpiączka”** w tym znaczeniu oznacza brak reakcji na bodźce słowne lub stymulacje fizyczną, przy czym odruchy mogą lecz nie muszą być obecne. Głębokość śpiączki może ulegać zmianie w ciągu doby.

© Nota praw autorskich

Narzędzie Palliative Performance Scale wersja 2 (PPSv2) jest objęte prawami autorskimi Victoria Hospice Society. Zastępuje ono jego pierwszą wersję opublikowaną w 1996 r [J Pall Care 9 (4): 26-32]. Narzędzie nie może być modyfikowane lub wykorzystywane do innych celów niż opisane powyżej. Programy mogą wykorzystywać PPSv2 z odpowiednią adnotacją. Prośby o udostępnienie w formacie elektronicznym Word na adres judy.martell@caphealt.org. Korespondencję należy wysyłać do Medical Director, Victoria Hospice Society, 1900 Fort St, Victoria, BC, V8R 1J8, Canada
